# Dexamethasone Administration During Late Gestation Has No Major Impact on Lipid Metabolism, but Reduces Newborn Survival Rate in Wistar Rats

**DOI:** 10.3389/fphys.2018.00783

**Published:** 2018-07-03

**Authors:** Katia Motta, Patricia R. L. Gomes, Paola M. Sulis, Silvana Bordin, Alex Rafacho

**Affiliations:** ^1^Multicenter Postgraduate Program in Physiological Sciences, Laboratory of Investigation in Chronic Diseases, Department of Physiological Sciences, Center of Biological Sciences, Federal University of Santa Catarina, Florianópolis, Brazil; ^2^Department of Physiology and Biophysics, Biomedical Sciences Institute, University of São Paulo, São Paulo, Brazil

**Keywords:** dexamethasone, glucocorticoid, glucose homeostasis, lipid tolerance, newborn survival, pregnancy, triacylglycerol

## Abstract

A rise in plasma triacylglycerol levels is a common physiological occurrence during late gestation and excess of glucocorticoids (GCs) has been shown to impair lipid metabolism. Based on those observations, we investigated whether the administration of dexamethasone during the late gestational period could exacerbate this pregnancy associated hypertriacylglycerolemia in rats. For this, female Wistar rats were treated with dexamethasone (0.2 mg/kg of body mass in the drinking water on days 14–19 of pregnancy; DP group) or equivalent days in the virgin rats (DV group). Untreated pregnant rats (control pregnant group) and age-matched virgin rats (control virgin group) were used as controls. Functional, biochemical, and molecular analyses were carried out after treatment with GC and in the control groups. Euthanasia was performed on day 20 of pregnancy. The metabolic parameters of the mothers (dams) at the time of weaning and 6 months later, as well as newborn survival, were evaluated. We observed that neither dexamethasone nor pregnancy affected blood glucose or glucose tolerance. Hypertriacylglycerolemia associated with lipid intolerance or reduced hepatic triacylglycerol clearance was observed during the late gestational period. GC treatment caused a further increase in basal plasma triacylglycerol levels, but did not have a significant effect on lipid tolerance and hepatic triacylglycerol clearance in pregnant rats. GC, but not pregnancy, caused few significant changes in mRNA expression of proteins involved in lipid metabolism. Dexamethasone during pregnancy had no impact on lipid metabolism later in the dams’ life; however, it led to intra-uterine growth restriction and reduced pup survival rate. In conclusion, GC exposure during the late gestational period in rats has no major impact on maternal lipid homeostasis, soon after parturition at weaning, or later in the dams’ life, but GC exposure is deleterious to the newborn when high doses are administered at late gestation. These data highlight the importance of performing an individualized and rigorous control of a GC treatment during late pregnancy considering its harmful impact on the fetuses’ health.

## Introduction

The first two-thirds of gestation are characterized by an anabolic condition that includes hyperphagic behavior, which provides energy for optimal embryo/fetus development (Zeng et al., 2017). This anabolic phase shifts to a catabolic state in the last third of gestation in order to sustain a faster fetal growth (Herrera and Ortega-Senovilla, 2014). This last period is characterized by a rise in plasma triacylglycerol as a result of various mechanisms that include: (i) an increased breakdown of lipid stores that exports more non-esterified free fatty acids into circulation (Alvarez et al., 1996; Catalano et al., 2006), (ii) a reduction in lipoprotein lipase (LPL) activity in peripheral tissues resulting in decreased triacylglycerol clearance from circulating lipoproteins (Alvarez et al., 1996), and (iii) increased production and release of hepatic very low-density lipoprotein (VLDL; Knopp and Zhu, 1997). These peripheral adaptations are partially explained by reduced insulin sensitivity and increased plasma estrogen levels that occur in this third gestational period both in rodents and women (Herrera and Ortega-Senovilla, 2014).

**GRAPHICAL ABSTRACT:**
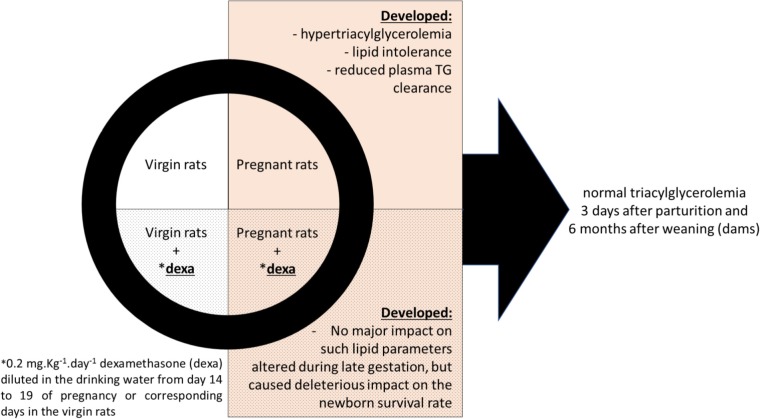
Effects of dexamethasone administration during the late gestational period on lipid metabolism.

Increased circulating triacylglycerol levels during the late gestational period are physiological and, in normal pregnancies, return to pre-pregnancy levels after delivery, as demonstrated in rodents (Argiles and Herrera, 1981) and in women (Wild et al., 2016). However, abnormal elevations in plasma triacylglycerol levels during pregnancy have been associated with gestational hypertension and preeclampsia, and may be related to preterm births and macrosomic babies (Vrijkotte et al., 2012). In humans, abnormal elevations in triacylglycerol levels during late gestation commonly correlate with excess weight and obesity (Farias et al., 2016), sedentarism (Monda et al., 2009), smoking (Gastaldelli et al., 2010), vitamin D deficiency (Başaranoğlu et al., 2017), and use of some medications (i.e., beta blockers, valproate, and sertraline; Wild et al., 2016) in mothers.

A considerable number of pregnant women suffer from chronic diseases that require glucocorticoid (GC) treatment. For instance, pregnant patients with asthma or rheumatoid arthritis depend on GC therapy during pregnancy (Gur et al., 2004; Namazy and Schatz, 2005). In addition, GCs may be prescribed during early pregnancy if a fetus with congenital adrenal hyperplasia is present (Van Vliet et al., 2008) or during late gestation if there is a risk of preterm delivery (Forhead and Fowden, 2011; Singh et al., 2012).

Unfortunately, there may be undesirable effects of these medications. Studies in pregnant rats, nonhuman primates, and humans have shown that maternal administration of synthetic GC (i.e., dexamethasone and betamethasone) during late pregnancy may induce intra-uterine growth restriction (IUGR) and post-natal cardiometabolic abnormalities such as hypertension and type 2 diabetes mellitus (Fowden et al., 1998; Khulan and Drake, 2012; Braun et al., 2013). However, the effects of GCs during late pregnancy on maternal metabolic outcomes, as well as later in the dams’ life are also not fully elucidated. Thus, in this study, we evaluated the impact of dexamethasone administration during the late gestational period on overall metabolism with emphasis on lipid metabolism. We hypothesized that dexamethasone administration, due to its diabetogenic actions (Pasieka and Rafacho, 2016), might exacerbate the physiological elevation in plasma triacylglycerol observed in the third period of gestation. Overall, we demonstrated that GC had no major impact on maternal lipid homeostasis during late pregnancy or later in the dams’ life, but impacted heavily on pup survival.

## Materials and Methods

### Ethical Approval

The experimental protocol was approved by the Federal University of Santa Catarina Committee for Ethics in Animal Experimentation (approval ID: PP00782) in accordance with the Brazilian National Council for Animal Experimentation Control (CONCEA).

### Materials

Dexamethasone phosphate (Decadron) was purchased from Aché (Campinas, Brazil). Poloxamer (P-407) was purchased from Sigma (St. Louis, MO, United States). The reagents used in the hepatic glycogen and fat content protocols, glucose tolerance test, histology, and adipose tissue lipolysis were acquired from LabSynth (Diadema, Brazil) and Sigma (St. Louis, MO, United States). Commercial olive oil used in the lipid tolerance test was purchased from Andorinha (Alferrarede, Portugal).

### Animals

A total of 204 female and 41 male Wistar rats were used in this study. The animals were supplied by the Federal University of Santa Catarina’s Animal Breeding Center, and transferred to a laboratory animal house facility where they were kept at 21 ± 2°C on a 12-h light–dark cycle (lights on at 0600). All rats had *ad libitum* access to food (commercial standard chow for rats, Nuvilab CR-1; Nuvital, Brazil) and filtered tap water.

### Experimental Design and Animal Treatment Protocols

Six-weeks-old nulliparous Wistar rats were acclimatized for a period of 6 weeks before being randomly assigned to one of four groups. Two groups of female rats were housed with male rats for up to 8 days (three females and one male per cage). The presence of spermatozoa in a vaginal lavage was considered as day 0 of gestation and this female rat was transferred to a separate cage. The average rate of pregnancy success was 75% (153 of 204 became pregnant; this is the average rate of success obtained in the Federal University of Santa Catarina’s Animal Breeding Center). The rats that did not become pregnant were relocated to other projects of the lab with protocols approved by the Federal University of Santa Catarina’s Committee for Ethics in Animal Experimentation. Pregnant rats were housed in individual cages from day 12 of gestation until parturition but remained in audio-visual and olfactory contact with other animals at all times. Another group of age-matched virgin rats was also housed individually in the same environment. One group of pregnant rats (DP group) and one of virgin rats (DV group) received daily GC (water-soluble dexamethasone) at a dose of 0.2 mg⋅kg^-1^⋅day^-1^ diluted in the drinking water from day 14 to 19 of pregnancy or corresponding days in the virgin rats. The dexamethasone dose was adjusted on a daily basis according to the water intake on the previous day, and adjusted to the body mass of the current day. The control rats [virgin and pregnant, control virgin (CV), and control pregnant (CP) groups, respectively] did not receive dexamethasone in the water. The dose of dexamethasone used was that reported on a previous publication using rats as an experimental model (Gomes et al., 2014).

**FIGURE 1:**
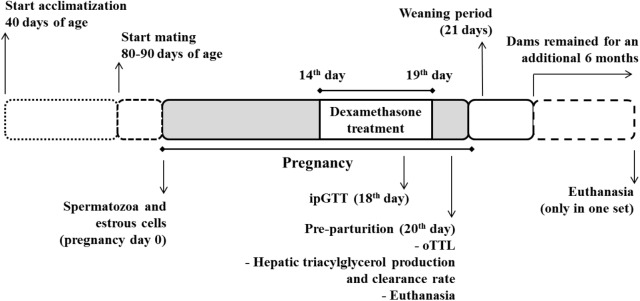
Experimental design. Pregnant rats were treated with dexamethasone (0.2 mg⋅kg^-1^⋅day^-1^ diluted in the drinking water) from the 14^th^ to the 19^th^ day of pregnancy. A parallel group of virgin rats (not shown) were submitted to the same treatment protocol on equivalent days. The pregnant and virgin rats not treated with dexamethasone (not shown) remained on drinking water *ad lib.*

Details of groups are depicted in **Figure 1**: (i) CV – virgin rats that received only filtered tap water *ad libitum*; (ii) CP – pregnant rats that received only filtered tap water *ad libitum*; (iii) dexamethasone-treated virgin (DV) – virgin rats that received dexamethasone diluted in the drinking water; and (iv) dexamethasone-treated pregnant (DP) – pregnant rats that received dexamethasone diluted in the drinking water. This design was repeated in five different sets of rats, labeled set 1, set 2, set 3, set 4, and set 5 that were assigned to the experiments described in **Table 1**. All experiments/methods were performed simultaneously on the four groups (CV, CP, DV, and DP). All pregnant rats in set 1 were observed until parturition occurred (to collect newborns and maternal data after delivery) and remained under observation during lactation and until 6 months after weaning their litter (**Figure 1**).

**Table 1:** Distribution of experiments by set of animals.

	Set 1	Set 2	Set 3	Set 4	Set 5
^1^Food intake and body mass	X	X	X	X	X
^2^Blood glucose on day 13 and day 20	X	X	X		
^2^Plasma TG on day 13	X	X	X		
^2^Plasma TG on day 20	X	X	X	^4^X	^4^X
Post-parturition data	X				
^3^Organs’ mass (euthanasia)		X		X	
Biochemical data (euthanasia)		X			
GTT during gestation			X		
Adipose tissue lipolysis			X		
PCRs			X		
Adipocyte morphometry			X		
LTT during gestation				X	
Hepatic TG release and clearance index					X
Hepatic glycogen and TG content	X		X		X


**FIGURE 2:**
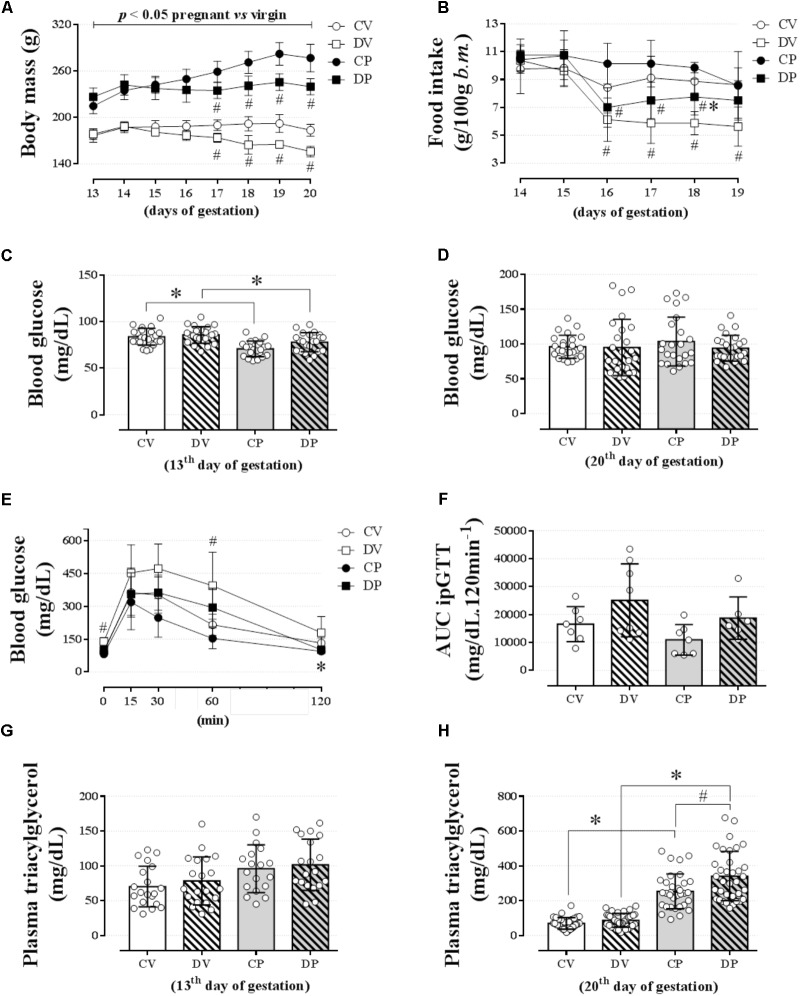
Body mass, food intake, blood glucose, glucose tolerance, and plasma triacylglycerol levels. The average body mass **(A)**; food intake **(B)**; blood glucose on the 13^th^ **(C)** and 20^th^ gestational day **(D)**; blood glucose values during an ipGTT (2 g/kg, body mass, *i.p.*) on the 18^th^ gestational day **(E)**; AUC for blood glucose values during ipGTT **(F)**; plasma triacylglycerol levels on the 13^th^ **(G)** and 20^th^ gestational day **(H)** in rats drinking 0.2 mg⋅kg^-1^⋅day^-1^ water-soluble dexamethasone diluted in the water from the 14^th^ to the 19^th^ day of pregnancy or equivalent days in virgin rats. Results are expressed as mean ± SD. ^∗^ and ^#^ indicate a significant difference compared to the respective control groups using ordinary two-way ANOVA with Tukey’s *post hoc* (*n* = 8–38/group, *p <* 0.05; see main text for detailed description). AUC, area-under-glucose-curve.

*^1^Assessed in all sets of animals, but only one representative set was used for graphical representation in **Figures 2A,B**.*

*^2^The numbers refer to the day of pregnancy.*

*^3^Included in this “*n*” were the aggregate samples from set 2 and set 4.*

*^4^These plasma TG values were taken from the baseline data obtained during LTT and hepatic TG release experiments (sets 4 and 5, respectively).*

*GTT; glucose tolerance test, LTT; lipid tolerance test; PCR; polymerase chain reaction; TG; triacylglycerol.*

### Metabolic *in Vivo* Measurements

Daily food intake was assessed from day 14 to 19 of pregnancy (or equivalent days in the virgin groups); chow consumption within a 24-h interval was normalized to total body mass. Body mass was measured daily from day 13 to 20 of pregnancy (or equivalent days in the virgin groups) and for a separate group of dams (set 1) every 3 days of lactation and at 2, 4, and 6 months after weaning (the same protocol was performed in age-matched virgin rats). When indicated, % change in body mass and food intake were determined according to the formula [(final value - initial value)/initial value]^∗^100. Blood samples in living rats were collected from the tip of the tail of fasted rats (12 h) for measures of blood glucose and plasma triacylglycerol levels on day 13 of pregnancy. Plasma triacylglycerol was also measured from blood sampled from the tip of the tail of the dams on days 3, 8, and 21 after parturition and 3 months after weaning or equivalent days in virgin rats (on rats from set 1). Blood glucose was determined from a drop of blood with a glucometer (Accu-Check Performa; Roche Diagnostics GmbH, Mannhein, Germany). Circulating triacylglycerol was determined in plasma obtained after blood (70 μl) centrifugation (400 × *g*) into EDTA-NaF-containing tubes (Glistab – Labtest; Lagoa Santa, Brazil) with enzymatic colorimetric assays (Biotecnica, Varginha, Brazil) according to the manufacturer’s instructions. Blood glucose determination on day 20 of pregnancy and 6 months after weaning (dams) or equivalent days in virgin rats (set 1) was also measured with a glucometer in a drop of blood collected from the tip of the tail immediately before euthanasia. Determination of plasma triacylglycerol on day 20 of pregnancy and 6 months after weaning (dams) or equivalent days in virgin rats (set 1) was done in plasma obtained from trunk blood collected into EDTA-NaF-containing tubes after euthanasia (by exposure to a CO_2_-rich atmosphere followed by decapitation).

### Intraperitoneal Glucose Tolerance Test (ipGTT)

The intraperitoneal glucose tolerance test (ipGTT) was performed on day 18 of pregnancy (or equivalent day in virgin rats) in fasted (12 h) rats. The rats had the tip of the tail cut for blood collection. The first drop was discarded, and the second drop was used for the determination of blood glucose (time 0) using a glucometer as described before. A 50% glucose solution pre-warmed at 36°C (2 g/kg, body mass) was immediately administered by intraperitoneal (*i.p.*) injection, and blood samples collected from the tail tip at 15, 30, 60, and 120 min for blood glucose measurements (Battiston et al., 2017). ipGTT was also performed in fasted dams 6 months after weaning or equivalent days in virgin rats on the day of euthanasia (set 1). Area-under-glucose-curve (AUC) was obtained after normalization by the initial value.

### Oral Lipid Tolerance Test (oLTT)

The oral lipid tolerance test (oLTT) was performed on day 20 of pregnancy (or equivalent day in virgin rats) in fasted (12 h) rats. Baseline (time 0) plasma triacylglycerol was measured on a 70 μl blood sample as described in section “Metabolic *in Vivo* Measurements.” Commercial olive oil (5 ml/kg, body mass) was immediately administered via oral gavage, and blood samples were collected from the tail tip at 60, 120, 180, and 240 min for plasma triacylglycerol measurements as described in section “Metabolic *in Vivo* Measurements” and elsewhere (Merola et al., 2011). AUC was obtained after normalization by the initial value.

### Hepatic Triacylglycerol Production and Clearance Rate

Hepatic triacylglycerol production and clearance rate were measured on day 20 of pregnancy (or equivalent day in virgin rats) in fasted (12 h) rats. Baseline (time 0) plasma triacylglycerol was measured on a 70 μl blood sample as described in “Metabolic *in Vivo* Measurements.” A poloxamer (P-407)-containing solution (1 g/kg, body mass, *i.p.*) diluted in saline 0.9% (Millar et al., 2005) was immediately administered. Blood samples were collected from the tail tip at 60, 120, 180, and 240 min for determination of plasma triacylglycerol. P-407 was dissolved overnight and kept in ice before administration in order to maintain the polymer in a mobile viscous state for the injection. The rate of hepatic triacylglycerol release was calculated using the formula (final plasma triacylglycerol – baseline plasma triacylglycerol)/240 (the accumulation time in min) and normalized to 100 g of body mass. Plasma clearance was the ratio between the normalized hepatic triacylglycerol release and the baseline plasma triacylglycerol concentration (Millar et al., 2005; Oakes et al., 2005).

### Euthanasia and Biochemical Data

All rats were euthanized and trunk blood collected as described in section “Metabolic *in Vivo* Measurements.” The plasma was separated and stored at -80°C until later determination of insulin, total cholesterol, and high-density lipoprotein (HDL)-cholesterol. Plasma insulin was quantified by AlphaLISA technology (Perkin Elmer, Waltham, MA, United States – cat. no. AL204) following the manufacturer’s instructions and previously published data (Battiston et al., 2017). Enzymatic colorimetric assay for the quantification of cholesterol and HDL-cholesterol from Biotécnica (Varginha, Brazil) was performed according to the manufacturer’s instructions. Organs (listed in “Results” section) were carefully removed and weighed. Visceral fat from omental, retroperitoneal, and perigonadal depots was collected. Determination of insulin sensitivity by the homeostatic model assessment (HOMA-IR) was based on the following formula: [fasting glycemia (mM) × fasting insulinemia (μU/ml)]/22.5 according to Battiston et al. (2017). Four sets of animals (sets 2, 3, 4, and 5) were euthanized on day 20 of pregnancy; their pups were euthanized by decapitation. A separate set of animals was allowed to deliver undisturbed and the pups were counted and weighed at the end of the day of delivery (postnatal day 1) or on the following day (if delivery had occurred during the lights off period; postnatal day 2). The dams were kept with litters of eight pups each (four female and four male); the extra pups were euthanized by decapitation. As will be described in section “Results,” 100% of the offspring from dexamethasone-treated dams died within 72 h after delivery. After weaning of offspring from dams, all dams (control and dexamethasone-treated) were rehoused five animals per cage and continued under observation for an additional 6 months. Dams previously treated with dexamethasone were kept in their individual cages during an equivalent period of lactation as that of control dams to avoid the influence of adult rat density per cage on the data.

### Liver Glycogen and Hepatic Triacylglycerol Content

Hepatic glycogen content was determined according to a previous publication (Rafacho et al., 2007). Hepatic glycogen was determined by a phenol-based assay using a spectrophotometer. Determination of hepatic triacylglycerol content was performed according to a previous publication (Gonçalves-Neto et al., 2014). The hepatic triacylglycerol content was determined by an enzymatic colorimetric method according to the manufacturer’s instructions (Biotecnica).

### Adipose Tissue Lipolysis

Adipose tissue lipolysis was performed according to previous publications (Nunes et al., 2013; Motta et al., 2015) immediately after euthanasia. *Ex vivo* adipose tissue lipolysis was assayed by incubating tissue samples (the same amount for all animals) and measuring glycerol release into the incubation medium. Perigonadal adipose tissue samples (100 mg) were incubated in aerated (5% CO_2_: 95% O_2_) Krebs buffer (pH 7.4) containing 1% bovine serum albumin for 60 min at 37°C. After incubation, samples were collected and kept in ice. Glycerol concentrations were determined by an enzymatic colorimetric assay as described before for plasma triacylglycerol assessment.

### Adipocyte Morphometry

Samples of perigonadal adipose tissue (the same amount for all animals) were collected on the day of euthanasia and transferred to a histological plastic cassette. Samples were fixed in a 4% paraformaldehyde solution for 48 h at 8°C, dehydrated in ethanol, and embedded in paraffin. Representative tissue sections (5 μm) were obtained using a hand microtome and placed on individual glass slides. Then, the sections were dewaxed in xylol and rehydrated. Finally, the sections were stained with hematoxylin and eosin and coverslipped using Canada’s balsam. The stained sections were scanned using the Axio Scan slide scanner (ZEISS, Oberkochen, Germany) and ZenLite software (Blue edition, ZEISS, Oberkochen, Germany) was used for determination of morphometric parameters. Cell perimeter was obtained by the manual contouring of cells with intact plasma membranes. Ten to fifteen fields (approximately 350 cells per section) were randomly analyzed for determination of adipocyte mean perimeters. Perimeter distribution was ranked according to predefined ranges in small, medium, large, and X-large (Strakovsky et al., 2015).

### RNA Extraction and qPCR

Total RNA was extracted from samples of liver tissue and perigonadal adipose tissue (∼100 mg) collected from fasting animals using Trizol reagent (Invitrogen, Carlsbad, CA, United States). Extracted RNA was diluted in RNase-free water according to the manufacturer’s specifications and quantified by spectrophotometry at 260 nm with acceptable 260/280 nm ratios between 1.8 and 2.0. RNA quality was assessed by ethidium bromide agarose gel electrophoresis. For mRNA expression analysis, 1 μg of total RNA was reverse transcribed using Improm-II reverse transcriptase (Promega, Madison, WI, United States) and random primers, according to the manufacturer’s instructions. Real-time amplifications were performed using Kappa SYBR Fast DNA polymerase (Kappa Biosystems, Boston, MA, United States) standard procedures (Lellis-Santos et al., 2012). The primer sequences for RNA amplification are described in **Table 2**. All reactions were performed using 60°C for the annealing step of amplification. Values of mRNA expression were normalized to the internal control genes *Rpl37a*, *Rn18s*, and *Ppib* (cyclophilin) using the ΔΔC_T_ method.

**Table 2:** List of primer sets.

Gene	Forward primer (5’–3’)	Reverse primer (5’–3’)
*Ppib*	CTCCGTGGCCAACGATAAGA	AGGTCACTCGTCCTACAGGT
*Rn18s*	GAC TCA ACA CGG GAA ACC TCA CC	TCG CTC CAC CAA CTA AGA ACG G
*Rpl37a*	CAA GAA GGT CGG GAT CGT CG	ACC AGG CAA GTC TCA GGA GGT G
*Acaca*	TGCTTATATTGTGGATGGCTTG	TTCTACTGTCCCTTCTGGTTCC
*Apob*	CTGCGGTGGCAGAAATAACG	CCTTGAGCAAACCTTAGGTAGGG
*Dgat2*	AAGCCCATCACCACCGTTG	TTCCTTCCAGGAGCTGGCAC
*Fasn*	TGGTGAAGCCCAGAGGGATC	CACTTCCACACCCATGAGCG
*Foxo1*	CTCACACATCTGCCATGAACCG	GTCCATGAGGTCGTTCCGAATG
*Nr3c1*	TCTCCTCCATCCAGCTCGTCAGC	TGCAGCTTCCACATGTCAGCACC
*Dusp6*	TGTCCTGGTGCATTGCTTGG	GGTGAAGTAGAGCTGCTGTGCG
*Akt*	AGAGCGGGTGTTCTCTGAGGAC	GAAAGGCAACCTCCCACACATC
*Srebf1*	ACTGGTAGAGCACATTCCC	CAGTTGATGTAGAGGCTAAGC
*Mttp*	TATGACCGTTTCTCCAAGAGTGG	TCAAGGTTCTCCTCTCCCTCATC
*Lrp*	CCT ACC AAC TTC ACC AAC CCA G	GTT CTC GCT TCT CGT CCG TG
*Ldlr*	CTG CTG TGT CAC TGA AGC G	GTC ACC TTG GAC TTG GAA G


*All qRT-PCR primers were designed using Nucleotide BLAST (NCBI – National Center for Biotechnology Information), based on the standard criteria for the qRT-PCR primer design considering Tm, GC contents, primer length, and the number of CG dimers. These sets were developed and tested. The optimal primer sequences are shown.*

### Statistical Analysis

All analyses were performed using GraphPad Prism^®^ Version 6.01 software (GraphPad Inc., La Jolla, CA, United States). The results were expressed as the mean ± SD for parametric data or median and interquartile ranges for non-parametric data of the number (*n*) of animals. The symmetry of the data was tested by Kolmogorov–Smirnov, Shapiro–Wilk, and D’Agostino and Pearson omnibus normality tests. It was considered symmetric if approved by at least one of three tests. Analysis of variance (two-way ANOVA) followed by Tukey’s *post hoc* test was used for multiple comparisons of parametric data or Kruskal–Wallis followed by Dunn’s *post hoc* when the variables presented an asymmetric distribution. When indicated in the figure legend, paired or unpaired Student’s *t*-test and unpaired Mann–Whitney test were applied for parametric and non-parametric data, respectively. Extreme studentized deviate method was applied to determine whether a value had reached significant outlier (Grubb’s test, available online on GraphPad QuickCalcs). Reference in the text to “their respective control groups” means differences among CP and DP groups vs. CV and DV groups, respectively (effect of pregnancy, indicated with ^∗^), or DV and DP groups vs. CV and CP groups, respectively (effect of dexamethasone, indicated with ^#^). Significance was set at *p* < 0.05.

## Results

### Dexamethasone Further Increased the Elevation in Plasma Triacylglycerol Levels Observed in Late Gestation

Changes in body mass and food intake during pregnancy and the effect of dexamethasone between day 13 and 20 of pregnancy (or equivalent for virgins) are shown in **Figures 2A,B**, respectively. The body masses of pregnant and virgin control rats increased continuously, being the largest in pregnant rats (**Figure 2A**; *n* = 8/group; *p <* 0.05). Dexamethasone caused a significant reduction or even abolished the gain in body weight in virgin and pregnant rats, respectively, 3 days after start of dexamethasone treatment compared to their respective controls (**Figure 2A**; *n* = 8/group; *p* < 0.05). This was associated with a significant decrease in food intake in both dexamethasone-treated groups, which was more pronounced and persistent in virgin rats (**Figure 2B**; *n* = 7–8/group; *p <* 0.05). Percent changes in body mass and food intake from the beginning (day 14 of pregnancy) to the end of dexamethasone treatment (day 19 of pregnancy) are presented in **Table 3**.

**Table 3:** Body mass and food intake variation rate (%) from the beginning (day 14 of pregnancy) to the end of dexamethasone administration (day 19 of pregnancy).

	CV	DV	CP	DP
^1^Body mass	4.2 ± 2.0	-12.3 ± 1.2^#^	20.2 ± 3.4^∗^	1.6 ± 2.9^∗,#^
^1^Food intake	-7.2 ± 14.2	-42.8 ± 11.5^#^	-4.8 ± 9.1	-27.8 ± 10.4^#^


*^*1*^(% from day 14). Results are expressed as mean ± SD. Symbols ^∗^ and ^#^ indicate significant difference compared to the respective control groups using ordinary two-way ANOVA with Tukey’s *post hoc* test (*n* = 7–8/group, *p <* 0.05; see main text for a detailed description). *F*(1,27) = 270.80 and *F*(1,27) = 370.6 for pregnancy and dexamethasone effect in body mass, respectively. *F*(1,27) = 49.23 for dexamethasone effect in food intake.*

Pregnancy, but not dexamethasone, reduced fasting blood glucose on day 13 of gestation (1 day before the start of the dexamethasone treatment) in pregnant rats to the virgin controls [**Figure 2C**; *n* = 21–23/group, *F*(1,85) = 28.56; *p <* 0.05]. On day 20 of pregnancy (1 day after the end of dexamethasone treatment), all blood glucose values returned to normal as there was no longer any difference among all groups (**Figure 2D**; *n* = 23–25/group; NS). Neither pregnancy nor dexamethasone affected glucose tolerance, assessed by AUC data (**Figures 2E,F**, respectively; *n* = 6–8/group; NS), but DV rats exhibited higher glycemic values during baseline and 60 min after glucose load compared to the CV group (*p <* 0.05).

Pregnancy exerted a major impact on lipid metabolism. The fasting plasma triacylglycerol assessed on day 13 of pregnancy was similar among all groups (**Figure 2G**; *n* = 18–20/group; NS). However, on day 20 of pregnancy, the plasma triacylglycerol levels were significantly increased in pregnant rats compared to the virgin controls [**Figure 2H**; *n* = 32–38/group, *F*(1,136) = 208.3; *p <* 0.05]. Dexamethasone treatment during late gestation resulted in an additional incremental effect in plasma triacylglycerol levels [**Figure 2H**; *n* = 33–38/group, *F*(1,136) = 11.97 for dexamethasone effect and *F*(1,136) = 5.28 for interaction; *p <* 0.05].

### Dexamethasone During Late Pregnancy Had Minimal Impact on Lipid Tolerance and No Impact on Hepatic Export or Clearance of Triacylglycerol

**FIGURE 3:**
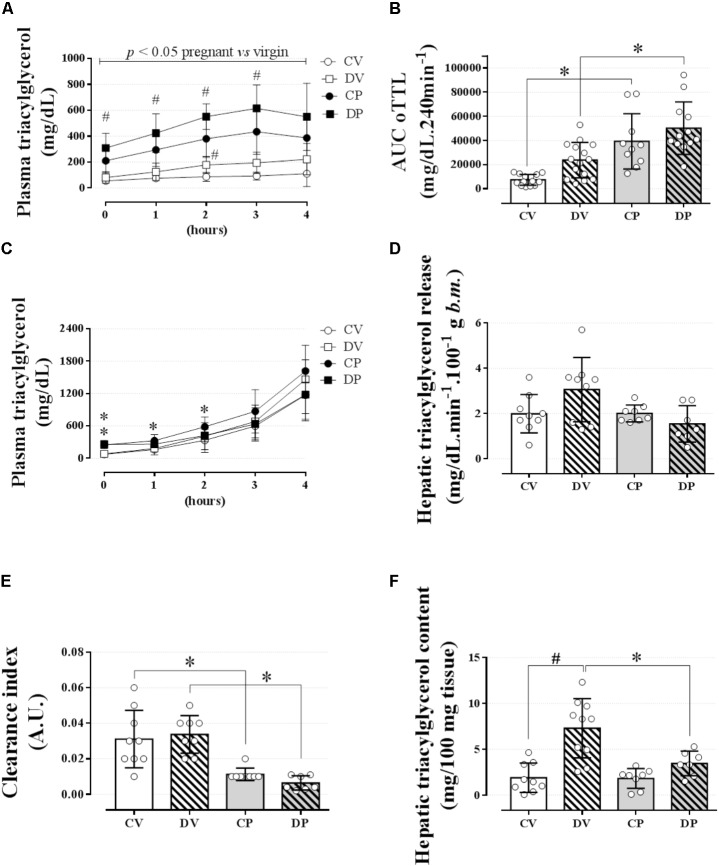
Lipid tolerance and hepatic lipid export. The plasma triacylglycerol values during an oLTT (5 ml/kg, body mass) on the 20^th^ gestational day **(A)**; AUC values for oLTT **(B)**; plasma triacylglycerol values during four consecutive hours after P-407 administration (1 g/kg, body mass) on the 20^th^ gestational day **(C)**. **(D)** Hepatic triacylglycerol release into circulation, **(E)** hepatic triacylglycerol clearance index, and **(F)** hepatic glycogen content immediately after the end of a 4-h experiment. Rats ingested 0.2 mg⋅kg^-1^⋅day^-1^ of water-soluble dexamethasone diluted in the water from 14^th^ to the 19^th^ day of pregnancy or equivalent days in virgin rats. Results are expressed as mean ± SD. ^∗^ and ^#^ indicate a significant difference compared to the respective control groups using ordinary two-way ANOVA with Tukey’s *post hoc* (*n* = 8–15/group, *p <* 0.05; see main text for detailed description). The differences at baseline include DP and CP vs. the respective controls, and in hours 1 and 2 only CP vs. CV. AUC, area-under-triacylglycerol-curve.

We next evaluated lipid tolerance and hepatic triacylglycerol export and clearance to investigate whether there is any involvement of the liver or other peripheral structures that could explain such elevation in the plasma triacylglycerol values in pregnant rats. Lipid intolerance was observed in both pregnant groups compared to their respective virgin groups (**Figure 3A**; *n* = 10–15/group; *p <* 0.05) and confirmed by the AUC data [**Figure 3B**; *n* = 10–15/group, *F*(1,47) = 37.8; *p <* 0.05]. Dexamethasone treatment increased plasma triacylglycerol levels in almost all data points measured in pregnant rats and at 2 h post oil load in virgin rats compared to untreated CV and pregnant rats (**Figure 3A**; *p <* 0.05). However, this increment in plasma triacylglycerol was not enough to promote significant lipid intolerance in dexamethasone-treated rats compared to their respective controls as shown by the AUC (**Figure 3B**). The accumulation of plasma triacylglycerol after inhibition of LPL with P-407 was the same in all groups after 3 or 4 hours (**Figure 3C**; *n* = 8–9/group; NS). Hepatic triacylglycerol release (normalized to body mass) revealed no significant change during late pregnancy or by GC treatment (**Figure 3D**; *n* = 8–9/group; NS). The plasma clearance index of triacylglycerol was significantly reduced in pregnant rats compared to the virgin controls [**Figure 3E**; *n* = 8–9/group, *F*(1, 28) = 40.40; *p <* 0.05]. Again, dexamethasone had no effect on this parameter. Hepatic triacylglycerol content assessed immediately after the end the experimental protocol was larger in the DV rats compared to their respective control group [**Figure 3F**; *n* = 8–9/group, *F*(1,30) = 7.21; *p <* 0.05].

**FIGURE 4:**
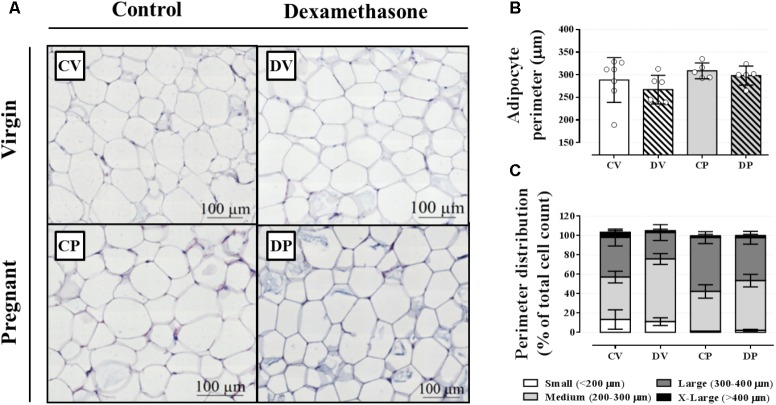
Adipocyte morphology and perimeter. Representative images of perigonadal adipose tissue **(A)**, average adipocyte perimeter **(B)**, and perimeter distribution ranges **(C)** in rats drinking 0.2 mg⋅kg^-1^⋅day^-1^ water-soluble dexamethasone diluted in the water from 14^th^ to the 19^th^ day of pregnancy or equivalent days in virgin rats. Results are expressed as mean ± SD in “**B**.” In “**C**,” the variance was expressed as standard error of the mean (SEM) for esthetic reasons (*n* = 5–10/group; see main text for detailed description). Sections were stained in hematoxylin and eosin.

We next evaluated the perigonadal adipocyte perimeter in order to investigate any association between an elevation in circulating triacylglycerol and adipocyte hyperplasia or hypertrophy in perigonadal white adipose tissue. The average adipocyte size, based on the adipocyte perimeter, was similar among all groups (**Figures 4A,B**; *n* = 5–7/group; NS). In order to further characterize adipose tissue morphology, adipocytes were classified according to four perimeter ranges: small (<200 μm), medium (200–300 μm), large (300–400 μm), and extra-large (>400 μm). Neither pregnancy nor dexamethasone led to a significant alteration in adipocyte size distribution (**Figures 4A,C**; *n* = 5–7/group; NS).

### Dexamethasone During Late Pregnancy Increased Circulating Insulin, Total Cholesterol, Hepatic Mass, or Hepatic Glycogen Content

In order to investigate whether some features of dexamethasone treatment that typically occur in virgin rats also occurred in pregnant rats, we measured several metabolic parameters on the day of euthanasia (**Table 4**). The relative perigonadal and omental masses were similar among all groups, except for the retroperitoneal mass, which was reduced in virgin rats after exposure to dexamethasone [*n* = 12–16/group, *F*(1,49) = 14.42; *p <* 0.05]. The relative mass of the adrenal glands was reduced in pregnant rats [*n* = 7–8/group, *F*(1,26) = 38.02; *p <* 0.05] whereas the relative spleen mass was decreased in dexamethasone-treated rats [*n* = 7–8/group, *F*(1,27) = 113.30; *p <* 0.05]. Dexamethasone treatment caused hyperinsulinemia, elevated total plasma cholesterol, plasma HDL-cholesterol and also albumin levels in both pregnant and virgin rats (*n* = 7–9/group; *p <* 0.05). Dexamethasone-treated rats also exhibited increased hepatic glycogen content and relative liver mass (*n* = 7–16/group; *p <* 0.05). Hepatic triacylglycerol content and basal glycerol release were not altered by dexamethasone nor pregnancy (*n* = 7–9/group; *p* < 0.05). HOMA index was increased in dexamethasone-treated rats (*n* = 7–9/group; *p <* 0.05).

**Table 4:** Organ masses and plasmatic and hepatic metabolic data on the day of euthanasia.

	CV	DV	CP	DP
^1^Liver	3.0 ± 0.3	3.7 ± 0.5^#^	3.0 ± 0.3	3.7 ± 0.3^#^
^1^Retroperitoneal fat	1.4 ± 0.4	0.9 ± 0.4^#^	1.5 ± 0.2	1.3 ± 0.3^∗^
^1^Perigonadal fat	1.8 ± 0.8	1.5 ± 0.5	1.8 ± 0.4	1.7 ± 0.5
^2^Omental fat	132 ± 55	146 ± 32	153 ± 34	144 ± 67
^1^Visceral fat	3.3 ± 0.8	2.6 ± 1.0	3.8 ± 1.0	3.0 ± 0.9
^2^Adrenal gland	35 ± 5.0	34 ± 5.0	28 ± 1.3^∗^	23 ± 2.0^∗^
^2^Spleen	240 ± 28	170 ± 16^#^	235 ± 28	122 ± 21^∗,#^
				
^3^Plasma insulin	0.15[0.07;0.25]	1.18[0.8;1.7]^#^	0.29[0.07;0.49]	1.48[1.2;1.6]^#^
^4^Total cholesterol	92[79;103]	156[125;185]^#^	121[103;143]	201[171;245]^#^
^4^HDL-cholesterol	61[48;74]	103[85;119]^#^	57[48;72]	93[82;104]^#^
^5^Plasma albumin	3.6 ± 0.4	4.4 ± 0.5^#^	3.5 ± 0.4	4.3 ± 0.3^#^
				
^6^Hepatic glycogen content	0.3[0.13;0.53]	2.7[2.4;3.0]^#^	0.4[0.20;0.72]	2.1[1.5;2.0]^#^
^6^Hepatic triacylglycerol content	8.0 ± 1.2	9.3 ± 3.2	5.2 ± 1.5	6.4 ± 2.0
^7^Basal glycerol release	5.6[3.5;7.8]	7.5[5.3;8.9]	2.2[2.0;6.7]	5.3[1.2;6.9]
^8^HOMA-IR	1.0[0.4;1.9]	8.7[5.0;7.0]^#^	1.6 ± [0.27;2.9]	10.0[8.5;11.5]^#^


*^*1*^(g/100 g *b.m.*); ^*2*^(mg/100 g *b.m.*); ^*3*^(ng/mL); ^*4*^(mg/dL); ^*5*^(g/dL); ^*6*^(mg/g tissue); ^*7*^(μg/g tissue.h^-*1*^); and ^*8*^see methods for details. Results are expressed as mean ± SD for ^*1,2,5,6*^(hepatic triacylglycerol) and median ± interquartile range for ^*3,4,6*^(hepatic glycogen)^*7,8*^ as they are asymmetrically distributed (nonparametric). ^∗^ and ^#^ indicate significant difference compared to the respective control groups using ordinary two-way ANOVA with Tukey’s *post hoc* test for ^*1,2,5,6*^(hepatic triacylglycerol) and Kruskal–Wallis with Dunn’s *post hoc* test for ^*3,4,6*^(hepatic glycogen)^*7,8*^ (*n* = 7–16/group, *p <* 0.05; see main text for a detailed description). b.m., body mass.*

### Dexamethasone Treatment but Not Pregnancy Exerts Minor Effect on the Expression Profile of Genes Related to Liver and Perigonadal Fat Lipid Metabolism

**FIGURE 5:**
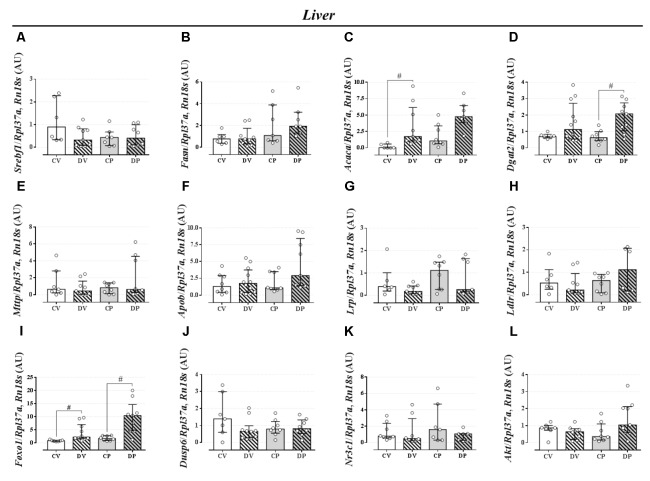
mRNA expression of lipid metabolism targets in the liver. Liver mRNA expression of *Srebf1* **(A)**, *Fasn* **(B)**, *Acaca* **(C)**, *Dgat2* **(D)**, *Mttp* **(E)**, *Apob* **(F)**, *Lrp* **(G)**, *Ldlr* **(H)**, *Foxo1* **(I)**, *Dusp6* **(J)**, *Nr3c1* **(K)**, and *Akt* **(L)** in rats drinking 0.2 mg⋅kg^-1^⋅day^-1^ water-soluble dexamethasone diluted in the water from 14^th^ to the 19^th^ day of pregnancy (DP) or equivalent days in virgin rats (DV). CV and CP are the virgin and pregnant control, not treated rats. Results are expressed as median ± interquartile range as they are asymmetrically distributed (nonparametric). ^#^ indicates a significant difference compared to the respective control groups using Kruskal–Wallis with Dunn’s *post hoc* test (*n* = 4–12/group, *p <* 0.05; see main text for detailed description).

**FIGURE 6:**
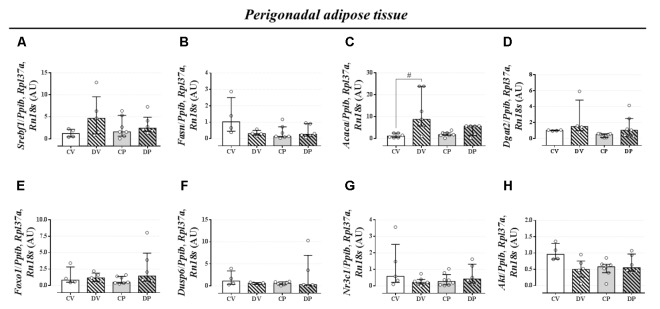
mRNA expression of lipid metabolism targets in the perigonadal tissue. Perigonadal mRNA expression of *Srebf1* **(A)**, *Fasn* **(B)**, *Acaca* **(C)**, *Dgat2* **(D)**, *Foxo1* **(E)**, *Dusp6* **(F)**, *Nr3c1* **(G)**, and *Akt* **(H)** in rats drinking 0.2 mg⋅kg^-1^⋅day^-1^ water-soluble dexamethasone diluted in the water from 14^th^ to the 19^th^ day of pregnancy (DP) or equivalent days in virgin rats (DV). CV and CP are the virgin and pregnant control, not treated rats. Results are expressed as median ± interquartile range as they are asymmetrically distributed (nonparametric). ^#^ indicates a significant difference compared to the respective control groups using Kruskal–Wallis with Dunn’s *post hoc* test (*n* = 4–12/group, *p <* 0.05; see main text for detailed description).

The expression of genes involved in the lipogenic pathway such as *Srebf1*, the gene that encodes the sterol regulatory element-binding protein 1 and *Fasn* that encodes the fatty acid synthase protein, was not altered by dexamethasone or pregnancy either in the liver (**Figures 5A,B**, respectively; *n* = 7–9/group; NS) or in the perigonadal adipose tissue (**Figures 6A,B**, respectively; *n* = 4–7/group; NS). However, the gene for *Acaca*, a gene that encodes acetyl CoA-carboxylase (ACC), was upregulated in virgin rats treated with dexamethasone both in the liver and in the perigonadal fat (**Figures 5C**, **6C**, respectively; *n* = 6–9/group; *p <* 0.05). *Dgat2*, a gene that encodes O-acyltransferase 2 isozyme, was also upregulated in the liver of DP rats (**Figure 5D**; *n* = 6–12/group; *p <* 0.05), but not in perigonadal fat (**Figure 6D**; *n* = 4–7/group; NS). The expression of two genes involved in VLDL assembly in the liver, genes that encode the microssomal triacylglycerol transfer protein (*Mttp*) and apolipoprotein B (*Apob*), were not altered by dexamethasone or pregnancy (**Figures 5E,F**, respectively; *n* = 7–12/group; NS). The expression of genes that encode two liver receptors, lipoprotein receptor-related protein (*Lrp*), and low-density lipoprotein receptor (*Ldlr*), was also not altered by dexamethasone treatment or pregnancy (**Figures 5G,H**, respectively; *n* = 7–9/group; NS).

The expression of *Foxo1* (forkhead box protein homeobox 1), a gene related to the regulation of gluconeogenesis, was upregulated in the liver of dexamethasone-treated rats (**Figure 5I**; *n* = 7–10/group; *p <* 0.05). The expression of the gene encoding hepatic mitogen-activated protein kinase phosphatase (MKP3, named as *Dusp6* gene) that dephosphorylates and activates FOXO1 protein was not altered by dexamethasone or pregnancy (**Figure 5J**; *n* = 8–10/group; NS). The hepatic GC receptor (GR, named *Nr3c1* gene) gene expression, a protein known to positively regulates MKP3 in the liver, remained unaltered by the effects of dexamethasone or pregnancy (**Figure 5K**; *n* = 7–8/group; NS). In perigonadal adipose tissue, the expression of these genes was not altered by dexamethasone or pregnancy (**Figures 6E–G**; *n* = 4–8/group; NS). Finally, gene expression for protein kinase B (PKB, named *Akt* gene) was not altered either in the liver or perigonadal fat (**Figures 5L**, **6H**, respectively; *n* = 4–7/group; NS).

### Dexamethasone Treatment During Late Pregnancy Did Not Cause Any Impact Later in the Dams’ Life, but Did Impact Pup Survival Rate

**FIGURE 7:**
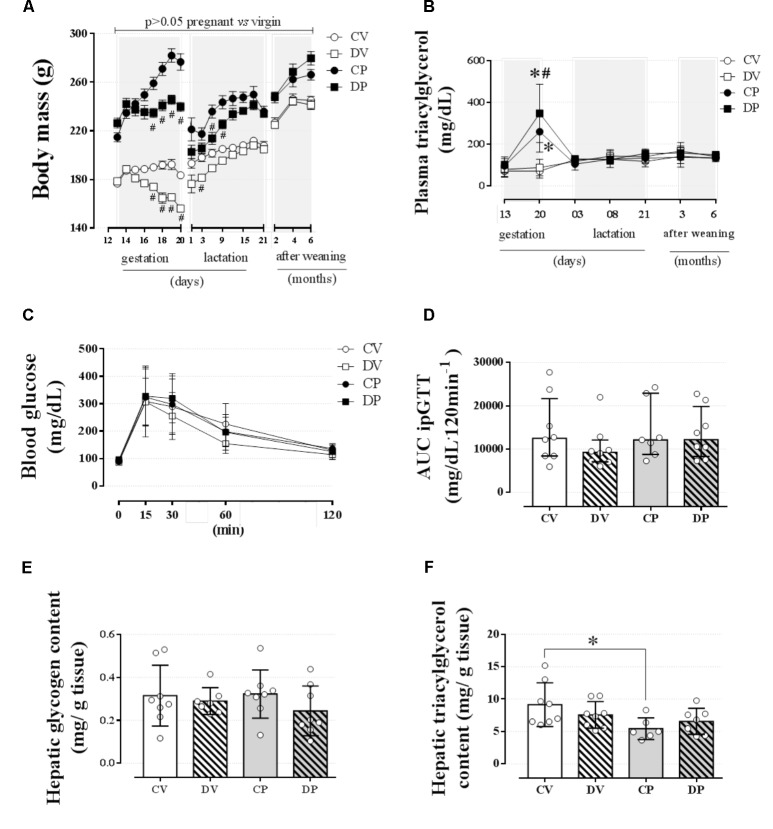
Metabolic status of dam rats 6 months after weaning. The average dam’s body mass **(A)**; dam’s plasma triacylglycerol at gestation, weaning, and after weaning **(B)** and blood glucose values during an ipGTT **(C)**; AUC for ipGTT blood glucose values **(D)**; dam’s hepatic glycogen content **(E)**; and hepatic triacylglycerol content **(F)** 6 months after weaning in rats drinking 0.2 mg⋅kg^-1^⋅day^-1^ water-soluble dexamethasone diluted in the water from 14^th^ to the 19^th^ day of pregnancy or equivalent days in virgin rats. Results are expressed as mean ± SD for “**B**,” “**C**,” “**E**,” and “**F**” and median ± interquartile range for “**D**” as they are asymmetrically distributed (nonparametric). In “**A**,” the variance was expressed as standard error of the mean (SEM) for esthetic reasons. ^∗^ and (^#^) indicate a significant difference compared to the respective control groups using ordinary two-way ANOVA with Tukey’s *post hoc* test for “**A**,” “**B**,” “**C**,” “**E**,” and “**F**” data and Kruskal–Wallis with Dunn’s *post hoc* test for “**D**” data (*n* = 6–8/group, *p <* 0.05). Data in “**A**” and “**B**” related to gestational period are the same as those shown in **Figures 2A,H**, respectively. AUC, area-under-glucose-curve.

In order to verify whether dexamethasone treatment during late gestation could impair the dam’s metabolism later in life, we assessed some parameters on the third and sixth months after the end of weaning. Interruption of dexamethasone administration that coincided with the end of gestation or equivalent days in virgin rats resulted in recovery of the body mass in the dams at weaning and 6 months after the end of the weaning period or equivalent days in virgin rats (**Figure 7A**; *n* = 7–8/group). Plasma triacylglycerol levels were completely normalized 3 days after delivery and remained unaltered for the entire of the remaining period of observation (**Figure 7B**; *n* = 7–8/group). Blood glucose and glucose tolerance remained unchanged in dams 6 months after the end of the weaning period or equivalent days in virgin rats (**Figures 7C,D**; *n* = 7–8/group; NS). Hepatic glycogen content returned to control values in DP and virgin rats (**Figure 7E**; *n* = 7–8/group; NS), but hepatic triacylglycerol content was decreased in CP dams compared to their virgin controls [**Figure 7F**; *n* = 6–8/group, *F*(1,25) = 6.71; *p <* 0.05].

**FIGURE 8:**
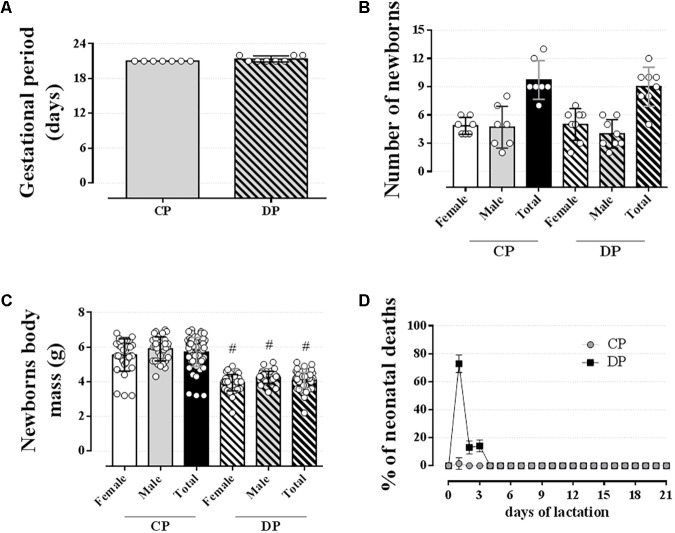
Newborns data. The average gestational period **(A)**, number of newborns **(B)**, newborns body mass **(C)**, and % of neonatal deaths **(D)** of mothers drinking 0.2 mg⋅kg^-1^⋅day^-1^ water-soluble dexamethasone diluted in the water from 14^th^ to the 19^th^ day of pregnancy. Results are expressed as mean ± SD. ^#^ indicates a significant difference compared to the respective control groups using ordinary two-way ANOVA with Tukey’s *post hoc* or unpaired Student’s *t*-test to compare the totals (*n* = 7–8/group for “**A** and **B**” and 32–39/group for “**C** and **D**,” *p <* 0.05).

Dexamethasone did not affect the gestational period or the total number of pups delivered (**Figures 8A,B**, respectively; *n* = 7–8 mothers/group; NS). However, dexamethasone administration during the late period of gestation promoted a significant reduction in the offspring body mass compared to the offspring from CP rats [**Figure 8C**; *n* = 32–39/group, *F*(1,100) = 126.20; *p <* 0.05]. Also, dexamethasone, at the dose used in this study, resulted in a 100% of offspring deaths within 3 days after delivery (**Figure 8D**; *n* = 32–39/group; *p <* 0.05).

## Discussion

A rise in plasma triacylglycerol levels is a common occurrence characteristic of the late period of gestation. Circulating levels of triacylglycerol increase throughout the second, and especially during the third trimester, and decrease after delivery, both in women (Knopp et al., 1986; Alvarez et al., 1996) and in rodents (Bosch and Camejo, 1967; Otway and Robinson, 1968; Argiles and Herrera, 1981). Depending on the dose and duration of treatment, GC-based therapies impact human and animal lipid metabolism, leading to increased triacylglycerol levels (reviewed by Peckett et al., 2011; Pasieka and Rafacho, 2016). In the present study, we observed that dexamethasone administration on the last 6 days of gestation in rats further increases the basal plasma triacylglycerol caused by pregnancy, but has no effects on lipid homeostasis later in the dams’ life. Moreover, functional tests (lipid tolerance, and hepatic lipid export and clearance assessed by inhibition of LPL) suggest that the elevated triacylglycerol levels found in pregnant rats are more likely due to a reduced clearance of plasma triacylglycerol than a result of increased hepatic triacylglycerol release or increased adipose tissue lipolysis.

Dexamethasone administration in the drinking water significantly reduced body mass in the virgin rats and prevented body mass gain in the pregnant rats (**Figures 2A,B** and **Table 3**). These effects were probably a direct consequence of the decreased food intake in both groups. The anorexic effect of dexamethasone, associated with a reduction in body mass, is in accordance with previous publications using male (Caperuto et al., 2006; Dos Santos et al., 2014) and female rats (Dos Santos et al., 2014; Battiston et al., 2017) treated with *i.p.* dexamethasone. Moreover, as shown in other studies, GC indirectly further increases levels of the anorexigenic hormones insulin and leptin (Caldefie-Chézet et al., 2001; Dos Santos et al., 2014; Battiston et al., 2017). These findings support our results in the dexamethasone-treated rats that had reduced body mass and food intake in association with hyperinsulinemia (**Table 4**). Regarding the pregnant rats, even though they probably had elevated actions of the anabolic/orexigenic pregnancy-related hormones (i.e., estradiol and progesterone), their body mass did not increase, but remained close to those before GC treatment; a fact that may have impaired the fetuses’ viability and survival.

Basal blood glucose and glucose tolerance were not modified by pregnancy or by dexamethasone during the end of gestational period (**Figures 2D,E**). The absence of any effect on glucose tolerance could be explained by the compensatory hyperinsulinemia observed in both groups. In the case of GCs, this is in accordance with previous studies in rats using dexamethasone at doses lower than 1.0 mg/kg body mass (a dose that causes glucose intolerance in male and female rats; Dos Santos et al., 2014; Battiston et al., 2017). This hyperinsulinemia is an indirect effect of GC on islet insulin hypersecretion, as demonstrated in male and female rats treated with dexamethasone (Novelli et al., 1999; Rafacho et al., 2008; Dos Santos et al., 2014). In the case of pregnancy, there is evidence for a reduced peripheral insulin sensitivity in maternal tissues that is associated with an increase of beta-cell mass, insulin synthesis, and glucose-stimulated insulin secretion in rats (Gomes et al., 2014) and women (Butler et al., 2010). Increased circulating prolactin and placental lactogen levels are responsible for the upregulation of endocrine pancreas function during pregnancy (Sorenson and Brelje, 1997). The adaptive endocrine pancreas compensations observed in rats are in part responsible for the maintenance of a normal glucose tolerance during these transitory metabolic insults during pregnancy and/or GC treatment.

Several studies have reported elevations in plasma triacylglycerol levels during pregnancy both in women (Knopp et al., 1986; Alvarez et al., 1996) and in rodents (Bosch and Camejo, 1967; Otway and Robinson, 1968; Argiles and Herrera, 1981). In our experiments with pregnant rats, we confirmed this metabolic phenotype and showed that it was further affected by dexamethasone administration (**Figure 2H**). As demonstrated by others, the elevation in triacylglycerol levels observed in late gestation may be a result of an increased rate of hepatic triacylglycerol export into the circulation, a decreased clearance of plasma triacylglycerol (Robinson, 1963), and/or an increase of adipose tissue glycerol release (Alvarez et al., 1996). Considering that dexamethasone treatment caused an impact on basal lipid metabolism, we decided to challenge lipid homeostasis with an oLTT. Lipid intolerance was observed in the pregnant rats, with no additional impact caused by GC during pregnancy (AUC in **Figure 3B**). This was probably a result of reduced adipose tissue LPL, the main enzyme responsible for plasma triacylglycerol clearance. In fact, there is evidence for a reduced adipose tissue LPL in late gestation in rats (Ramirez et al., 1983; Herrera, 2002) and women (Otway and Robinson, 1968; Ramirez et al., 1983). It should also be pointed out that the elevation in triacylglycerol levels during the lipid tolerance test in dexamethasone-treated rats (**Figure 3A**) was not enough to be translated into lipid intolerance in any of the treated groups (**Figure 3B**). This prandial elevation of triacylglycerol levels may contribute to the reduction in insulin sensitivity observed in dexamethasone-treated rats (**Table 4**), which in combination with pregnancy may help to explain the higher baseline values of plasma triacylglycerol in the DP group. Although plasma triacylglycerol levels are temporarily increased after a meal, this is not maintained for long periods, such as during basal metabolism (fasting) in virgin rats (**Figure 2H**), suggesting that pregnant-related factors interact with GC leading to elevated baseline values of plasma triacylglycerol.

Late pregnancy hypertriacylglycerolemia observed in our studies could have also been, at least in part, caused by a reduction of hepatic triacylglycerol clearance. However, this parameter was not affected by dexamethasone treatment. The absence of any alteration in adipose tissue glycerol release (**Table 4**) suggests a less relevant contribution of adipose tissue lipolysis to the hypertriacylglycerolemia seen in pregnant rats. In addition, the rate of triacylglycerol release into the circulation after treatment with P-407 revealed no contribution of such a mechanism as an explanation for the elevated levels of plasma triacylglycerol in pregnant rats, whether treated with GC or not (**Figures 3C,D**). This result was corroborated by the unaltered expression of genes that encode crucial enzymes involved in the lipogenic pathway and VLDL assembly (**Figures 5E,F**). Although dexamethasone administration was associated with upregulation of the hepatic *Dgat2*, we did not find any other alteration of genes related to triacylglycerol synthesis (i.e., *Srebf1* and *Fasn*) or VLDL assembly (i.e., *Mttp* and *Apob*). The clearance index data (**Figure 3E**), obtained after treatment with P-407, suggests a reduced ability of the liver of pregnant rats to remove circulating triacylglycerol, which was unaffected by dexamethasone. LPR and LDL-R proteins are two receptors expressed in the liver that are required for lipid metabolism and plasma protein clearance. However, there was no difference on *Lpr* or *Ldlr* mRNA expression in pregnant rats compared to controls (**Figures 5G,H**). Although these observations seem to be counterintuitive, these results are in agreement with a previous study in pregnant rats that showed normal expression of *Lpr* and *Ldlr* in late pregnancy (Smith et al., 1998).

We observed no major alterations of lipid homeostasis in DV rats, except for an increase in hepatic triacylglycerol content four hours after P-407 administration (**Figure 3F**). This increase was probably due to the upregulation of *Acaca* (ACC) mRNA in the liver (**Figure 5C**). ACC is an essential enzyme involved in *de novo* lipogenesis and is upregulated by GCs (Gathercole et al., 2011). This GC effect was somehow attenuated during pregnancy. These data indicate that the increased hepatic production of triacylglycerol in DV rats is retained in the liver and not released, which could avoid large oscillations in normal values of plasma triacylglycerol levels.

Other than the altered lipid homeostasis detected in pregnant rats, (i.e., elevated circulating triacylglycerol levels, lipid intolerance, and decreased plasma triacylglycerol clearance), there were no other major alterations caused by pregnancy. The e*x vivo* data obtained after euthanasia revealed some changes as a result of GC treatment that are in accordance with dexamethasone actions in rats, such as spleen atrophy (Rafacho et al., 2008; Dos Santos et al., 2014; Battiston et al., 2017), increased relative liver mass, decreased relative retroperitoneal fat mass, and increased hepatic glycogen content (Nunes et al., 2013; Gonçalves-Neto et al., 2014; Motta et al., 2015; **Table 4**). GCs can increase hepatic glycogen content by increasing glycogen synthase activity, as previously described (De Wulf and Hers, 1967). Our dexamethasone-treated rats (virgin and pregnant) had increased mRNA expression of *Foxo1* in the liver (**Figure 5I**) that is known to positively regulate the gluconeogenic pathway in the liver (Oh et al., 2013).

The circulating levels of total cholesterol and HDL-cholesterol were also elevated in rats treated with dexamethasone which could be partially explained by the genomic action of GR on the Apo1 promoter, as demonstrated previously in a study in humans involving treatment with oral prednisone 0.35 mg⋅kg^-1^⋅day^-1^ for 14 days (Ettinger and Hazzard, 1988). Apo1 upregulation leads to increased HDL-cholesterol synthesis and secretion. It is important to emphasize that reduced insulin sensitivity observed on dexamethasone-treated rats (**Table 4**), occurred independently of increased visceral adiposity (as evidenced by unchanged masses of adipose tissue depots – **Table 4**) reinforcing the negative impact of GCs in peripheral insulin sensitivity (Pasieka and Rafacho, 2016). Since this reduction in insulin sensitivity was not associated with major glucose and lipid metabolism alterations in pregnant and virgin rats, we suggest that the hyperinsulinemia could also be related to reduced activity of hepatic insulin-degrading enzymes seen in rats treated with dexamethasone (Protzek et al., 2016).

The high levels of triacylglycerol observed in late gestation returned to baseline in dams at the onset of lactation and remained unaltered up to 6 months after weaning (**Figure 7B**). This process was independent from the presence of pups since newborns from dexamethasone-treated dams died within 3 days after birth (**Figure 8D**). The return of the circulating triacylglycerol levels to baseline values was previously demonstrated in rats (Otway and Robinson, 1968; Agius et al., 1981) and women (Knopp et al., 1986; Desoye et al., 1987). These studies suggest that immediately after delivery maternal hypertriacylglycerolemia declines to pregestational values as a result of a decreased hepatic release of triacylglycerol into the circulation (Agius et al., 1981) and by increased LPL activity in the mammary glands that shifts plasma triacylglycerol to be used for milk synthesis (Otway and Robinson, 1968). Candidate hormones responsible for this normalization include a decline in plasma progesterone and a subsequent increase in circulating prolactin levels (Amenomori et al., 1970; Desoye et al., 1987). It is important to highlight that if the dexamethasone-treated dams had been able to suckle and care for their offspring, there may have been differences in metabolism that are not apparent in the absence of such metabolic demands.

Glucocorticoids had no long-term impact on body mass gain, lipid (i.e., plasma triacylglycerol levels and hepatic triacylglycerol content), and glucose metabolism (i.e., glucose tolerance and hepatic glycogen content) later in the dams’ life (up to 6 months after the end of weaning). Although our results showed no major effects of GC on overall lipid and glucose metabolism during late gestation and later in the dams’ life or equivalent days in virgin rats (**Figure 7**), the consequences of dexamethasone administration during pregnancy were severely deleterious to the pups. Dexamethasone treatment did not affect the duration of gestation or the number of newborns (**Figures 8A,B**), but resulted in IUGR, as demonstrated by others (Reinisch et al., 1978; Kauffman et al., 1994; Emgård et al., 2007). Studies with dexamethasone treatment for a shorter period during gestation (i.e., gestational days 14 and 15 or 17 and 19) also resulted in pups’ death (∼20%; Kauffman et al., 1994; Ozdemir et al., 2003). The deleterious effects of fetal exposure to synthetic GCs are reasonably well described and include, besides IUGR, deficits in organ development (i.e., kidneys, brain, and heart; Nyirenda et al., 1998; Singh et al., 2012) as well as reduced fetal–neonatal adrenergic mechanisms required to preserve cardiac function (Kauffman et al., 1994). We have no plausible explanation for the pups’ deaths, but we have evidence that maternal care is reduced on post-natal day 1 (Cella et al., unpublished results), which could favor hypothermia, undernutrition, and hypoglycemia in these pups, a question that merits investigation. We cannot rule out the fact that since some of the body mass of pregnant rats includes the weight of the pups, the pregnant female rats were actually getting a higher dose of dexamethasone than the virgin rats, and therefore could also have a negative impact on fetuses. It is important to point out that our findings do not intend to negate the use of GCs as a therapeutic maneuver in cases of risk of preterm delivery since there are well-established clinical conducts in such context. Nevertheless, we stress the need for great care to be taken when this therapy is applied to prevent further damaging the fetus.

## Conclusion

Except for basal plasma triacylglycerol levels, lipid intolerance and reduced triacylglycerol clearance from the circulation observed during late gestation is not worsened by treatment with dexamethasone. Thus, we conclude that GC exposure during late pregnancy in rats has no major impact on maternal lipid homeostasis or later in the dams’ life, but is deleterious to the newborns when in excess. These data highlight the importance for an individual and rigorous control of GC management during late pregnancy considering the harmful impact on the fetus.

## Data Availability

The datasets generated to support the findings of this study are available from the corresponding author upon reasonable request.

## Author Contributions

AR, KM, and SB contributed to the experimental design. KM, PS, and PG conducted the experiments. AR, KM, and PG contributed with analytic tools and data analysis and performed the data collection and analysis. AR and SB supplied reagents and materials. AR and KM wrote the paper. AR, KM, PG, and SB contributed to the discussion of the experimental findings. All authors read and approved the manuscript’s final format.

## Conflict of Interest Statement

The authors declare that the research was conducted in the absence of any commercial or financial relationships that could be construed as a potential conflict of interest.
